# Effect of acute administration of *Pistacia lentiscus *L. essential oil on rat cerebral cortex following transient bilateral common carotid artery occlusion

**DOI:** 10.1186/1476-511X-11-8

**Published:** 2012-01-12

**Authors:** Marina Quartu, Maria P Serra, Marianna Boi, Giuliano Pillolla, Tiziana Melis, Laura Poddighe, Marina Del Fiacco, Danilo Falconieri, Gianfranca Carta, Elisabetta Murru, Lina Cordeddu, Antonio Piras, Maria Collu, Sebastiano Banni

**Affiliations:** 1 Department of Biomedical Sciences, University of Cagliari, Cittadella Universitaria, Monserrato, Italy; 2 Department of Chemical Sciences, University of Cagliari, Cittadella Universitaria, Monserrato, Italy; 3 Nutrisearch srl, Pula, Italy

**Keywords:** Bilateral common carotid artery occlusion, reperfusion, DHA; COX-2, PEA, OEA, *Pistacia lentiscus *L., cerebral cortex, Wistar rat

## Abstract

**Background:**

Ischemia/reperfusion leads to inflammation and oxidative stress which damages membrane highly polyunsaturated fatty acids (HPUFAs) and eventually induces neuronal death. This study evaluates the effect of the administration of *Pistacia lentiscus *L. essential oil (E.O.), a mixture of terpenes and sesquiterpenes, on modifications of fatty acid profile and endocannabinoid (eCB) congener concentrations induced by transient bilateral common carotid artery occlusion (BCCAO) in the rat frontal cortex and plasma.

**Methods:**

Adult Wistar rats underwent BCCAO for 20 min followed by 30 min reperfusion (BCCAO/R). 6 hours before surgery, rats, randomly assigned to four groups, were gavaged either with E.O. (200 mg/0.45 ml of sunflower oil as vehicle) or with the vehicle alone.

**Results:**

BCCAO/R triggered in frontal cortex a decrease of docosahexaenoic acid (DHA), the membrane highly polyunsaturated fatty acid most susceptible to oxidation. Pre-treatment with E.O. prevented this change and led further to decreased levels of the enzyme cyclooxygenase-2 (COX-2), as assessed by Western Blot. In plasma, only after BCCAO/R, E.O. administration increased both the ratio of DHA-to-its precursor, eicosapentaenoic acid (EPA), and levels of palmytoylethanolamide (PEA) and oleoylethanolamide (OEA).

**Conclusions:**

Acute treatment with E.O. before BCCAO/R elicits changes both in the frontal cortex, where the BCCAO/R-induced decrease of DHA is apparently prevented and COX-2 expression decreases, and in plasma, where PEA and OEA levels and DHA biosynthesis increase. It is suggested that the increase of PEA and OEA plasma levels may induce DHA biosynthesis via peroxisome proliferator-activated receptor (PPAR) alpha activation, protecting brain tissue from ischemia/reperfusion injury.

## Background

Cerebral ischemia or stroke is often associated to unilateral or bilateral occlusion of internal carotid artery or common carotid artery [1-4]. Interruption of brain circulation makes the cerebral tissue unable to sustain basal metabolism and within a few seconds leads to multiple interconnected pathophysiological events affecting the structural and functional organization of brain tissue [5,6].

Although reperfusion of neural ischemic tissue is desirable, the post-ischemic reestablishment of blood supply leads to a general impairment of translation capability, oxidative stress and free radical formation which can eventually cause neuronal death [6-8]. Neuronal membranes are particularly rich in highly polyunsaturated fatty acids (HPUFAs) which serve as reservoirs of biologically active lipids in physiological conditions, whereas, in stressful circumstances, are target of free radical-mediated lipid peroxidation whose products, in turn, can injure the brain [9-11]. The brain has a unusual ability to preserve the neuronal membrane concentration of HPUFAs within a physiological range [12]; however, it is also particularly vulnerable to oxidative stress due to the presence of uniquely high content of HPUFAs, of an inadequate antioxidant defense system and of high mitochondrial density [10,11]. Ischemia/reperfusion may affect lipid turnover and cause the release of free (unesterified) PUFAs from membrane phospholipids [9]. The main products of lipid peroxidation are fatty acid hydroperoxides which are quite unstable and capable to propagate free radical reactions, thus extending the damage [13].

Interestingly, recent studies point up the relationship between brain metabolic stress and lipid peripheral dysregulation, showing that cleavage and immediate release of membrane lipid precursors, such as eicosanoids, could be useful indicators of central nervous system (CNS) pathologies [14]. In addition, plasma lipid metabolic alterations as a physiological response to brain ischemic stimulus are triggered by ischemia/reperfusion during carotid endoarterectomy in patients with carotid stenosis [15] and endocannabinoid (eCB) plasma levels have been shown to increase in patients with acute ischemic stroke [16].

Epidemiological studies indicate that oxidative stress and neuroinflammation are also factors associated with the decline of function in the aging brain [17] and that the regular consumption of natural products rich in bioactive compounds is associated with vascular diseases, stroke and dementia risk reduction [18-22]. Although the mechanisms through which these compounds exert beneficial effects wait to be clarified, their antioxidant, free radical scavenging and anti-inflammatory properties are supported by different experimental data [19,21-25]. The *Pistacia lentiscus *L. plant is an aromatic bush indigenous to Italy and other Mediterranean and Middle East countries. Its extracts have found extensive use in folk medicine for their anti-hypertensive, anti-inflammatory and antiseptic properties and for the treatment of gastric disorders [26]. To our knowledge, the bioactive properties of *P. lentiscus *L. extracts and/or individual components have been reported only by in vitro studies where, with different analytical methods, it has been shown that they have a strong antioxidant activity [25,27-33]. Previous analyses of *P*. *lentiscus *L. essential oil (E.O.) showed a characteristic composition with relatively high concentration of terpenes and sesquiterpenes [27-33], for some of which a potent anti-inflammatory activity has been shown in different experimental rodent models [21,22,34-36].

Because of the low extraction yield from *P. lentiscus *L. plant, the E.O. under study has not generally received much attention for its application in pharmaceutics and has little commercial use. We sought to investigate the potentially neuroprotective *in vivo *effects of dietary E.O., obtained by Supercritical Fluid Extraction (SFE), in a rat model of hypoperfusion achieved by transient bilateral common carotid artery occlusion (BCCAO) followed by reperfusion (BCCAO/R).

This study reports on modifications of fatty acid profile and eCB congener concentrations induced by BCCAO/R in the rat frontal cortex and plasma, and shows that lipid metabolic changes are apparently prevented by the administration of E.O.. Expression of the enzyme cyclooxygenase-2 (COX-2) is also examined by Western Blot on the same brains.

## Methods

### Extraction of *P. lentiscus *L. essential oil

#### Plant material

Leaves of *P. lentiscus *L. were collected during full blossom in northern Sardinia (Italy).

#### Reagents and standards

Highest purity solvents and reagents included Folin-Ciocalteu reagent, methanol, hydrochloric acid (37%), ferric chloride, sodium acetate trihydrate and sodium acetate 6-hydrate (Merck, Darmstadt, Germany); 1,1-Diphenyl-2-picrylhydrazyl radical (DPPH, 98%) and gallic acid (99%) (Sigma-Aldrich, Steinheim, Germany); sodium carbonate anhydrous and 2,4,6-tris(2-pyridyl)-S-triazine (TPTZ, P99%) (Fluka, Buchs, Germany); glacial acetic acid (SDS, Penien, France); dichloromethane (Lab-Scan, Dublin, Ireland). Chemical constituents of the oil were identified by comparison with reference compounds [Fluka, Acros Organics (Geel, Belgium); Sigma-Aldrich].

#### Preparation of the extracts

Supercritical CO_2 _(SFE) extractions were performed in a laboratory apparatus, equipped with a 320 cm^3 ^extraction vessel and two separator vessels of 300 and 200 cm^3^, respectively, connected in series [37]. Experiments were carried out at P = 90 bars, T = 50°C and ϕ_CO2 _= 0.6 kg/h. In the first separator the temperature was set at -10°C and the pressure at the same value as the extraction section. The second separator was set at 10°C and 15 bars. Extraction was carried out in a semi batch mode: batch charging of vegetable matter and continuous flow solvent. About 180 g of material were charged in each run.

#### Analysis

Analysis of essential oil was carried out by gas chromatography (GC) and by gas chromatography-mass spectrometry (GC-MS). Analytical GC was carried out in an Agilent 6890 gas chromatograph (Agilent Technologies, Palo Alto, CA, USA) with HP GC ChemStation data handling system, equipped with a single injector and two flame ionization detectors (FID). A Graphpak divider (Agilent Technologies) was used for simultaneous sampling to two Supelco fused silica capillary columns (Supelco Inc., Bellefonte, PA, USA) with different stationary phases: SPB-1 (polydimethylsiloxane 30 m × 0.20 mm I.D., film thickness 0.20 μm) and SUPELCOWAX 10 (polyethylene glycol 30 m × 0.20 mm I.D., film thickness 0.20 μm). Oven temperature was settled at 70°C, raising at 3°C min^-1 ^to 220°C and then held 15 min at 220°C; injector temperature: 250°C; carrier gas: helium, adjusted to a linear velocity of 30 cm/s; splitting ratio 1:40; detector temperature: 250°C.

GC-MS analyses were carried out in an Agilent 6890 gas chromatograph fitted with a HP1 fused silica column (polydimethylsiloxane 30 m × 0.25 mm I.D., film thickness 0.25 μm), interfaced with an Hewlett Packard mass selective detector 5973 (Agilent Technologies) operated by Agilent Enhanced ChemStation software. GC parameters as above; interface temperature: 250°C; MS source temperature: 230°C; MS quadrupole temperature: 150°C; ionization energy: 70 eV; ionization current: 60 μA; scan range: 35-350 u; scans/sec: 4.51.

The identity of the components was assigned by comparison of mass spectra and retention indices for two different chromatographic stationary phases calculated by linear interpolation to the retention of a series of *n*-alkanes. Experimental data were compared with corresponding data of reference oils and commercial available standards banked at a home-made library or from literature data [38,39]. Percentages of individual components were calculated based on GC peak areas without FID response factor correction.

Composition of the E.O. is reported in Table 1. The total essential oil yield, after an extraction lasting 4 hours, was 0.14% and the main constituents included germacrene D (19.9%), beta-caryophyllene (BCP) (6.6%), alpha-pinene (6.3%), myrcene (3.9%), beta-phellandrene (3.7%) and alpha-humulene (2.4%).

**Table 1:** Retention indices, *I*_R _and chromatographic area percentages (%) of compounds found in E.O. extracted by SFE at 90 bar, 50°C from *Pistacia lentiscus *L. leaves

**n**.	I_k_	Compound	%
1	922	tricyclene	-

2	926	alpha-thujene	0.2

3	933	alpha-pinene	6.3

4	948	camphene	0.3

5	973	sabinene	5.8

6	977	beta-pinene	2.4

7	991	myrcene	3.9

8	1006	alpha-phellandrene	1.5

9	1016	alpha-terpinene	0.4

10	1024	para-cymene	0.8

11	1028	beta-phellandrene	3.7

12	1046	(E)-beta-ocimene	-

13	1054	isopentil n-butanoate	0.2

14	1057	gamma-terpinene	0.7

15		n.i.	0.2

16	1088	terpinolene	0.2

17	1091	2-nonanone	0.2

18		n.i.	0.1

19	1101	linalool	0.2

20	1106	isopentyl isovalerate	-

21	1177	terpin-4-ol	1.0

22	1190	alpha-terpineol	0.2

23	1250	n.i.	0.4

24	1253	n.i.	0.2

25	1285	bornyl acetate	-

26	1294	2-undecanone	1.0

27	1302	n.i.	-

28	1349	alpha-cubebene	0.2

29	1375	alpha-copaene	1.7

30	1389	beta-cubebene	1.1

31	1391	beta-elemene	1.3

32	1408	n.i.	-

33	1418	beta-caryophyllene	6.6

34	1428	beta-gurjunene	0.2

35	1436	n.i.	0.7

36	1449	n.i.	0.3

37	1452	alpha-humulene	2.4

38	1459	allo-aromadendrene	0.9

39	1461	cis-muurola-4(14),5-diene	-

40	1473	trans-cadina-1(6),4-diene	-

41	1477	gamma-muurolene	2.9

42	1481	germacrene D	19.9

43	1485	beta-selinene	-

44	1490	trans-muurola-4(15),5-diene	0.3

45		n.i.	1.9

46	1495	alpha-selinene	1.7

47	1499	alpha-muurolene	1.6

48	1503	n.i.	0.9

49	1506	n.i.	-

50	1509	beta-bisabolene	1.1

51	1513	gamma-cadinene	8.7

52		n.i.	0.2

53	1524	delta-cadinene	4.2

54	1531	trans-cadina-1(2)-4-diene	0.5

55	1536	alpha-cadinene	-

56	1543	n.i.	0.4

57	1548	elemol	0.4

58	1555	n.i.	0.3

59	1557	elemicin	0.4

60	1564	(E)-nerolidol	-

61		n.i.	0.7

62	1575	spatutenol	1.8

63	1581	caryophyllene oxide	0.2

64	1583	n.i.	0.2

65	1589	n.i.	0.2

66	1613	n.i.	-

67	1615	n.i.	-

68	1626	n.i.	-

69	1630	gamma-eudesmol	-

70	1641	epi-alpha-muurolol	1.0

71	1645	alpha-muurolol	0.4

72	1648	beta-eudesmol	-

73	1653	alpha-cadinol	0.9

74	1682	epi-alpha-bisabolol	0.7

75	1684	alpha-bisabolol	0.3

76	1694	n.i.	3.1

### Experimental procedure

#### Animals and keeping

Sixteen male Wistar rats (Harlan-Italy, Udine, Italy ), weighing 210 ± 20 g (mean ± SD) were housed in an environment of controlled temperature (21 ± 2°C), relative humidity (60 ± 5%) and under an artificial 12 h light/dark cycle for 1 week before the experiment set off. All stressful stimuli were avoided. Animal care and handling throughout the experimental procedures were in accordance with the European Communities Council Directive of 24 November 1986 (86/609/EEC). The experimental protocols were also approved by the Animal Ethics Committee of the University of Cagliari. Standard laboratory food (Safe A04, France) and water were freely available *ad libitum*.

Animals were starved for 12 hours before surgery and 6 hours prior to ischemia the E.O. was administered by gavage. Due to the lack of data in the literature on the use of E.O. *in vivo*, choice of the dose was based on the concentration of some of the E.O. components, such as BCP and alpha-humulene (Table 1), whose effectiveness as single agents has been reported [34-36]. Since these molecules have been shown to be active in contrasting the symptoms of different models of inflammation in the range of 5 to 100 mg/kg bodyweight [34-36], in the present study each rat was administered 200 mg of E.O. (containing about 60 mg/kg bodyweight of BCP and 22 mg/kg bodyweight of alpha-humulene) in 0.45 ml of sunflower oil as vehicle. Rats were randomly assigned to four groups, those submitted to BCCAO/R and the sham-operated ones, treated with the vehicle alone or with the vehicle containing the E.O..

#### Surgery

Surgery was performed in all cases between 15:00 and 17:30 p.m.. Rats were anesthetized with intraperitoneal administration of chloral hydrate (80 mg/ml; 0.5 ml/100 g bodyweight). After a midline cervical incision and blunt dissection of muscles, the right and left common carotid arteries (CCA) were exposed while leaving the vagus nerve intact. Transient bilateral ligation of CCA, performed with a 0.28 mm nylon fishing line, lasted 20 min. Blood flow was then restored through the stenosed vessels for 30 min. Sham operated rats underwent a surgical procedure similar to the other groups but without the CCA occlusion. These animals made up the control group used to determine the effects of anaesthesia and surgical manipulation on the results.

#### Sampling

At the end of the procedure, the animals, still anesthetized, were sacrificed and different brain areas, i.e. olfactory bulb, telencephalic cortex (subdivided in an anterior third, including the frontal cortex, and the remaining posterior two thirds, including parietal, temporal, and occipital cortex), hippocampus, hypothalamus, striatum, brainstem and cerebellum were rapidly dissected out and frozen at - 80°C until fatty acid or western blot analysis. Blood was collected from the trunk of killed rats into heparinised tubes and centrifuged at 1500 g for 10 min at - 8°C. The resulting plasma was frozen at - 20°C until assayed for lipids.

### Lipid extraction

#### Measurement of fatty acid composition

Total lipids were extracted from different brain areas and plasma using chloroform/methanol 2:1 (v/v) [40]. Aliquots were mildly saponified as previously described [41] in order to obtain free fatty acids for high-performance liquid chromatography (HPLC) analysis. Separation of fatty acids was carried out with an Agilent 1100 HPLC system (Agilent, Palo Alto, Calif., USA) equipped with a diode array detector as previously reported [42].

#### Analysis of eCBs and congeners

N-arachidonoylethanolamide (AEA), 2-arachidonoyl-monoacylglycerol (2-AG), palmitoylethanolamide (PEA) and oleoylethanolamide (OEA) were extracted from tissues and plasma, purified and analysed as previously described [43].

One-way ANOVA and the Bonferroni test for post hoc analyses were applied to evaluate statistical differences among groups.

### Western blot

Tissue homogenates were prepared in 2% sodium dodecyl sulphate (SDS). Protein concentrations were determined using the Lowry method of protein assay [44] with bovine serum albumin as standard. Proteins for each tissue homogenate (30 μg), diluted 1:1 in loading buffer, were heated to 95°C for 10 min and separated by SDS-polyacrilamide gel electrophoresis (SDS-PAGE) using a 12.5% (w/v) polyacrylamide resolving gel. Internal mw standards (Kaleidoscope Prestained Standards, Bio-Rad, Hercules, CA, USA) were run in parallel. Two gels at a time were run for Coomassie staining and immunoblotting, respectively. Proteins for immunoblotting were electrophoretically transferred on a polyvinylidene fluoride membrane (Bio-Rad) using the Mini Trans Blot Cell (Bio-Rad). Blots were blocked by immersion in 20 mM Tris base and 137 mM sodium chloride (TBS) containing 5% milk powder and 0.1% Tween 20 (TBS-T), for 60 min at room temperature and incubated overnight at 4°C with rabbit polyclonal antibody against COX-2 (residues 570-598) (Cayman Chem., USA), diluted 1:200 in TBS containing 5% milk powder and 0.02% sodium azide (NaN3) (Sigma-Aldrich, Steinheim, Germany), was used as primary antiserum. After TBS-T rinse, blots were incubated for 60 min, at room temperature, with peroxidase-conjugated goat anti-rabbit serum (Sigma Aldrich), diluted 1:10,000 in TBS/T. Loading controls for equal loading of the wells were obtained by stripping and immunostaining the membranes as above, using a mouse monoclonal antibody against the housekeeping protein glyceraldehyde 3-phosphate dehydrogenase (GAPDH) (Chemicon), diluted 1:1,000, as primary antiserum, and a peroxidase-conjugated goat anti-mouse serum (Chemicon), diluted 1:5,000, as secondary antiserum. In order to control for non specific staining, blots were stripped and incubated with the relevant secondary antiserum. After TBS-T rinse, protein bands were visualized on a film (Kodak X-Omat LS, Kodak, Rochester, NY) using the ECL method (Amersham Corp.). Approximate molecular weight (mw) of immunolabelled protein bands and relative optical densities (ODs) were quantified using a GS-800™ Calibrated Densitometer with Quantity One 1 analysis software (BIO-RAD Hercules, CA, USA) by comparing the position of relevant bands on the autoradiograms with those of the prestained standard mw or with those of the GAPDH bands, respectively. The ratio of the intensity of COX-2 bands to the intensity of GAPDH bands was used to compare expression levels of these proteins following E.O. administration. One-way ANOVA and Fisher's test for post hoc analyses were applied to evaluate statistical differences among groups.

## Results

### Fatty acid and eCB profiles in brain tissue

**Figure 1:**
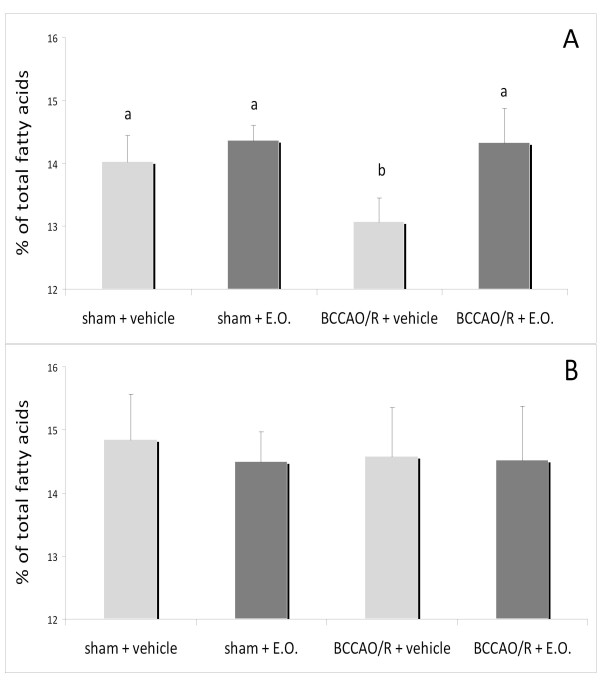
**DHA concentration, expressed as mol% of total fatty acids, in frontal (A) and posterior cortex (B) of rats submitted to sham operation and to BCCAO/R, pre-treated either with the vehicle alone or with the E.O.**. Error bars depict standard deviation. Different letters denote significant differences (p < 0.05).

Analysis of the frontal cortex (Figure 1A) and the remaining posterior cortex (Figure 1B) showed that, in vehicle-treated rats, BCCAO/R affected fatty acid concentration by causing about 10% decrease of DHA in the frontal cortex (Figure 1A). No changes were detected in the remaining posterior cortex (Figure 1B). By contrast, no significant changes were evident in rats pre-treated with E.O. and submitted to BCCAO/R as compared with other groups (Figure 1). Analysis of eCBs and congeners revealed no changes either after BCCAO/R or E.O. pre-treatment (data not shown).

### Fatty acid and eCB profiles in plasma

**Figure 2:**
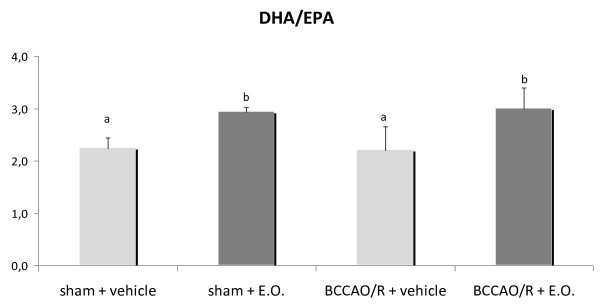
**DHA/EPA ratio in plasma of rats submitted to sham operation and to BCCAO/R, pre-treated either with the vehicle alone or with the E.O.**. Error bars depict standard deviation. Different letters denote significant differences (p < 0.05).

**Figure 3:**
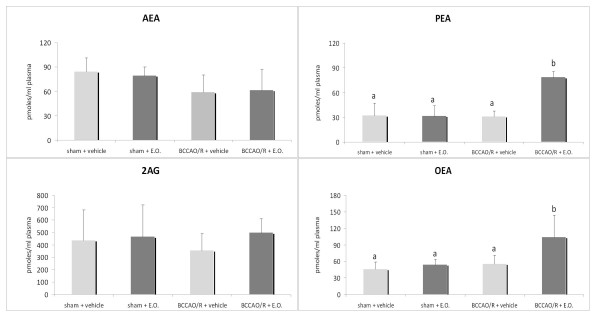
**AEA, PEA, OEA and 2-AG concentrations, expressed as pmoles/ml, in plasma of rats submitted to sham operation and to BCCAO/R, pre-treated either with the vehicle alone or with the E.O.**. Error bars depict standard deviation. Different letters denote significant differences (p < 0.05).

In order to verify whether there was a peripheral supply of DHA, we also measured levels of DHA and its precursor EPA in plasma. In rats pre-treated with E.O. whether or not submitted to BCCAO/R, an increase in the DHA-to-EPA ratio (p < 0.05) was observed (Figure 2). Analysis of eCBs and congeners revealed that levels of PEA and OEA increased significantly in rats pre-treated with E.O. and submitted to BCCAO/R (Figure 3) compared to the vehicle-fed ones, whereas no change was observed in levels of AEA and 2-AG.

### Western blot

**Figure 4:**
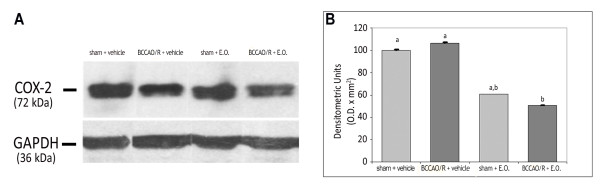
**(A)Western blot analysis of COX-2 in the frontal cortex of rats submitted to sham operation and to BCCAO/R, pre-treated either with the vehicle alone or with the E.O. (B)** Relative levels of COX-2 expression with densitometric analysis of the gray levels expressed as a percentage of the optical density (O.D.) ratio of the COX-2-positive bands to the GAPDH-positive ones. Error bars depict standard deviation. Different letters denote significant differences (p < 0.05).

In the frontal cortex homogenate, the staining intensity of the COX-2 protein band decreased significantly (p < 0.05) after pre-treatment with E.O. in the rats submitted to BCCAO/R, as compared with the vehicle-fed rats submitted to BCCAO/R (Figure 4). By contrast, no changes were observed between vehicle-fed rats, both after BCCAO/R or after sham operation.

## Discussion

The present results show that BCCAO for 20 min followed by reperfusion for 30 min is apt to induce a weak but reproducible insult which resulted in a decrease of DHA and a parallel increase of COX-2 expression in frontal cortex, without any appreciable change in tissue morphology. This model has proved to be useful to test E.O. effects in the early phase of BCCAO/R, before other factors may intervene triggering a range of physiological responses to preserve structural lipid changes.

Previous studies reported failure of this model to achieve a reproducible ischemic insult in the rodent brain due to the presence of efficient collateral systems, which allow for a cerebral blood flow compensation within a few minutes [45,46]. Results obtained show that this model triggers a cerebral insult sufficient to cause a detectable, significant decrease in the tissue level of DHA, the most abundant essential fatty acid of neuronal membrane phospholipids [47]. DHA is particularly susceptible to lipid peroxidation [48,49] and, for this reason, potentially apt to contribute to hypoperfusion/reperfusion-induced oxidative stress. However, several lines of evidence have associated increased levels of DHA to tissue protection in neuroinflammation [50] suggesting that DHA does not increase susceptibility to oxidative stress. Data in rodents and healthy humans support this inferring, as dietary supplementation with n-3 PUFAs does not affect lipid peroxidation [51,52]. DHA is an agonist of peroxisome proliferator-activated receptor (PPAR) alpha [53]. Though mechanisms certainly need further investigation, it can be suggested that PPARalpha, in turn, may act to attenuate the neuroinflammatory reaction either by increasing the rate of peroxisomal beta-oxidation or by slowing/suppressing nuclear factor-kappaB (NF-kB) activity and thereby slowing the activity of phospholipases and COX-2 [54].

The present findings demonstrate that just a single dose of E.O. is sufficient to influence tissue metabolism in response to BCCAO/R challenge. The BCCAO/R-induced decrease of DHA, most likely due to its breakdown during lipid peroxidation, was totally prevented by the pre-treatment with E.O.. The observation that DHA/EPA ratio raises after BCCAO/R following pre-treatment with E.O. may lead to suggest that there is an increased metabolism of n-3 which may supply damaged brain tissue with newly produced DHA. On the other hand, the increase of DHA/EPA ratio might be explained by an augmented entry of EPA in the brain parenchyma to be promptly metabolized to DHA [55,56]. Thus, in keeping with the protecting role of DHA in neuroinflammation [50], it is tempting to speculate that pre-treatment with E.O. affects the outcome of the BCCAO/R-induced events by modulating the tone of DHA precursors in the brain. In this way, the sparing of DHA from lipid peroxidation may contribute to minimize the extent of the BCCAO/R-induced inflammatory reaction and to help preserving the brain structure. The lower expression of COX-2 detected after BCCAO/R following pre-treatment with E.O. compared to the treatment with the vehicle alone further supports this hypothesis. In fact, upregulation of COX-2 mRNA and protein, though not always predictive of harmful effects [57-60], is induced by ischemia and plays a role in the ischemic brain injury [59,61-65]. Conversely, selective COX-2 inhibition ameliorates brain damage and prevents neuronal death after ischemia [66].

An increase of eCB plasmatic levels has been shown in several experimental models of ischemia [67]. In addition, in human stroke patients, an increase of PEA plasmatic levels in early phases of ischemia has been demonstrated to be significantly correlated with neurological disability [16]. In the present study PEA and OEA are increased only in BCCAO/R rats pre-treated with E.O.. Whether and how E.O. administration may trigger an increase of PEA and OEA plasma levels remains to be elucidated. As it has been suggested that brain damage "spill over" effect may contribute to eCB and congeners plasma levels [16], it would be further interesting to establish if E.O. may cause or modulate brain "spill over" in these conditions. On the other hand, it has been widely demonstrated that PEA has important pharmacological effects in a wide range of experimental models of inflammation [68-70]. In particular, it is possible that PEA may exert neuroprotective effects by preventing mast cell degranulation [71], activating PPARalpha [72] and dumping COX-2 expression [68]. It could be speculated that the augmentation of peripheral PEA might represent the key factor for enhancing PUFA metabolism via PPARalpha activation, protecting brain from ischemia/reperfusion injury. Interestingly, exogenous administration of PEA in an acute stroke model is effective in reducing the infarct size [73]. Moreover, in human stroke eCBs and congeners become detectable in relation to the acute phase of ischemic stroke [16] where they may play a role through multiple potential mechanisms [16].

## Conclusions

Previous studies tested the anti-oxidant, anti-microbial and anti-inflammatory activities of the *Pistacia lentiscus *L. gum or fixed oil [25,27,28,32,33]. However, to our knowledge this is the first report on the effect of *in vivo *administration of *Pistacia lentiscus *L. essential oil. Therefore, identification of the E.O. constituent(s) that may exert the beneficial effect in cerebral hypoperfusion/reperfusion remains an issue worth further investigations. A possible candidate is BCP, present in relatively high concentration in the E.O., which has been shown to elicit anti-inflammatory effects typical of non-steroidal agents in rat models of inflammation [34-36] and to exert cannabimimetic effects in vivo [20,21]. In addition, potential synergistic effects may be produced by other E.O. components, such as alpha humulene, germacrene D, gamma-cadinene, present in relatively high concentrations in E.O., for which antioxidant and antinflammatory activities have been also demonstrated [20,25,35].

Treatment with E.O. may prevent early neuroinflammatory events by protecting brain tissue DHA from oxidative degradation, possibly allowing an attenuation of signal transduction pathways which lead to increased susceptibility to neuroinflammation. This effect may be mediated by synergistic actions at peripheral and cerebral tissue level. Whether changes in brain and plasma fatty acid metabolism are directly interrelated to availability and modifications of PEA and OEA concentration in plasma is a suggestive issue worth to be further investigated.

## List of abbreviations

AEA: N-arachidonoylethanolamide; 2-AG: 2-arachidonoyl-monoacylglycerol; BCCAO/R: bilateral common carotid artery occlusion followed by reperfusion; BCP: beta-caryophyllene; COX-2: cyclooxygenase-2; DHA: docosahexaenoic acid; eCB: endocannabinoid; E.O.: *Pistacia lentiscus *L. essential oil; EPA: eicosapentaenoic acid; GAPDH: glyceraldehyde 3-phosphate dehydrogenase; HPLC: high-performance liquid chromatography; HPUFA: highly polyunsaturated fatty acid; NF-kB: nuclear factor-kappaB; OEA: oleoylethanolamide; PPAR: peroxisome proliferator-activated receptor; PEA: palmytoylethanolamide; PUFA: polyunsaturated fatty acid; SDS-PAGE: sodium dodecyl sulfate-polyacrilamide gel electrophoresis; SFE: Supercritical Fluid Extraction.

## Competing interests

The authors declare that they have no competing interests.

## Authors' contributions

MQ and SB conceived the study, participated in its design and coordination, draft and wrote the manuscript; MPS conceived and participated in the design of the study, carried out the protein analysis and elaborated the data; MB carried out the tissue sampling and performed the statistical analysis of western blot data; GP and TM performed the surgery; LP helped in the statistical analysis; MDF gave critical contribution to the manuscript; DF performed the SFE; GC and EM, participated in the design of the study, performed the eCB analysis and elaborated the data; LC and AP performed the fatty acid analysis and elaborated the data. All authors read and approved the final manuscript.

## References

[B1] Verhaeghe R Naert J Vermylen J Bilateral carotid artery occlusion: clinical presentation and outcome Clin Neurol Neurosurg 1991 93 123 126 10.1016/0303-8467(91)90052-Q 1652392

[B2] AbuRahma AF Copeland SE Bilateral internal carotid artery occlusion: natural history and surgical alternatives Cardiovasc Surg 1998 6 579 583 10.1016/S0967-2109(98)00072-6 10395259

[B3] Martin RS Edwards WH Mulherin JL Jr Edwards WH Jr Surgical treatment of common carotid artery occlusion Am J Surg 1993 165 3 302 306 10.1016/S0002-9610(05)80830-X 8447533

[B4] Persoon S Klijn CJ Algra A Kappelle LJ Bilateral carotid artery occlusion with transient or moderately disabling ischaemic stroke: clinical features and long-term outcome J Neurol 2009 256 1728 1735 10.1007/s00415-009-5194-3 19488672 PMC2758212

[B5] Dirnagl U Iadecola C Moskowitz MA Pathobiology of ischaemic stroke: an integrated view Trends Neurosci 1999 22 391 397 10.1016/S0166-2236(99)01401-0 10441299

[B6] White BC Sullivan JM DeGracia DJ O'Neil BJ Neumar RW Grossman LI Rafols JA Krause GS Brain ischemia and reperfusion: molecular mechanisms of neuronal injury J Neurol Sci 2000 179 S1-2 1 33 11054482 10.1016/s0022-510x(00)00386-5

[B7] Traystman RJ Kirsch JR Koehler RC Oxygen radical mechanisms of brain injury following ischemia and reperfusion J Appl Physiol 1991 71 1185 1195 1757340 10.1152/jappl.1991.71.4.1185

[B8] Shi H Liu KJ Cerebral tissue oxygenation and oxidative brain injury during ischemia and reperfusion Frontiers in Bioscience 2007 12 1318 1328 10.2741/2150 17127384

[B9] Bazan NG Synaptic lipid signaling: significance of polyunsaturated fatty acids and platelet-activating factor J Lipid Res 2003 44 2221 2233 10.1194/jlr.R300013-JLR200 13130128

[B10] Halliwell B Oxidative stress and neurodegeneration: where are we now? J Neurochem 2006 97 1634 1658 10.1111/j.1471-4159.2006.03907.x 16805774

[B11] Adibhatla RM Dempsey R Hatcher JF Integration of cytokine biology and lipid metabolism in stroke Frontiers in Bioscience 2008 13 1250 1270 10.2741/2759 17981627 PMC2083473

[B12] Neuringer M Connor WE Lin DS Barstad L Luck S Biochemical and functional effects of prenatal and postnatal omega 3 fatty acid deficiency on retina and brain in rhesus monkeys Proc Natl Acad Sci USA 1986 83 4021 4025 10.1073/pnas.83.11.4021 3459166 PMC323657

[B13] Yoshida Y Hayakawa M Habuchi Y Itoh N Niki E Evaluation of lipophilic antioxidant efficacy in vivo by the biomarkers hydroxyoctadecadienoic acid and isoprostane Lipids 2007 42 463 472 10.1007/s11745-007-3043-7 17476550

[B14] Centonze D Battistini L Maccarrone M The endocannabinoid system in peripheral lymphocytes as a mirror of neuroinflammatory diseases Curr Pharm Des 2008 14 23 2370 2342 10.2174/138161208785740018 18781987

[B15] Banni S Montisci R Sanfilippo R Finco G Sanna D Giordano E Murru E Cordeddu L Carta G Banni D Marchi A Physiological response to lipid peroxidation in ischemia and reperfusion during carotid endarterectomy Lipids Health Dis 2010 9 41 10.1186/1476-511X-9-41 20409338 PMC2874547

[B16] Naccarato M Pizzuti D Petrosino S Simonetto M Ferigo L Grandi FC Pizzolato G Di Marzo V Possible anandamide and palmitoylethanolamide involvement in human stroke Lipids Health Dis 2010 9 47 10.1186/1476-511X-9-47 20470384 PMC2877050

[B17] Ungvari Z Kaley G de Cabo R Sonntag WE Csiszar A Mechanisms of vascular aging: new perspectives J Gerontol A Biol Sci Med Sci 2010 65 1028 1041 20576649 10.1093/gerona/glq113 PMC2950814

[B18] Curin Y Ritz MF Andriantsitohaina R Cellular mechanisms of the protective effect of polyphenols on the neurovascular unit in strokes Cardiovasc Hematol Agents Med Chem 2006 4 277 288 10.2174/187152506778520691 17073605

[B19] Sun AY Wang Q Simonyi A Sun GY Botanical phenolics and brain health. Neuromolecular Med 2008 10 4 259 274 10.1007/s12017-008-8052-z 19191039 PMC2682367

[B20] Gertsch J Anti-inflammatory cannabinoids in diet: Towards a better understanding of CB(2) receptor action? Commun Integr Biol 2008 1 26 28 10.4161/cib.1.1.6568 19704783 PMC2633791

[B21] Gertsch J Leonti M Raduner S Racz I Chen JZ Xie XQ Altmann KH Karsak M Zimmer A Beta-caryophyllene is a dietary cannabinoid Proc Natl Acad Sci USA 2008 105 9099 9104 10.1073/pnas.0803601105 18574142 PMC2449371

[B22] Gertsch J Botanical drugs, synergy, and network pharmacology: forth and back to intelligent mixtures Planta Med 2011 77 1086 1098 10.1055/s-0030-1270904 21412698

[B23] Sousa OV Silvério MS Del-Vechio-Vieira G Matheus FC Yamamoto CH Alves MS Antinociceptive and anti-inflammatory effects of the essential oil from Eremanthus erythropappus leaves J Pharm Pharmacol 2008 60 6 771 777 18498714 10.1211/jpp.60.6.0013

[B24] Limem-Ben Amor I Boubaker J Ben Sgaier M Skandrani I Bhouri W Neffati A Kilani S Bouhlel I Ghedira K Chekir-Ghedira L Phytochemistry and biological activities of Phlomis species J Ethnopharmacol 2009 125 183 202 10.1016/j.jep.2009.06.022 19563875

[B25] Triantafyllou A Bikineyeva A Dikalova A Nazarewicz R Lerakis S Dikalov S Anti-inflammatory activity of Chios mastic gum is associated with inhibition of TNF-alpha induced oxidative stress Nutr J 2011 10 64 10.1186/1475-2891-10-64 21645369 PMC3127998

[B26] Atzei AD Le piante nella tradizione popolare della Sardegna 2003 Carlo Delfino Editore Sassari: Italy 221 222

[B27] Magiatis P Melliou E Skaltsounis AL Chinou IB Mitaku S Chemical composition and antimicrobial activity of the essential oils of *Pistacia lentiscus *var. chia. Planta Med 1999 65 8 749 752 10.1055/s-2006-960856 10630120

[B28] Baratto MC Tattini M Galardi C Pinelli P Romani A Visioli F Pogni R Antioxidant activity of galloyl quinic derivatives isolated from *Pistacia lentiscus *leaves Free Radical Research 2003 37 405 412 10.1080/1071576031000068618 12747734

[B29] Andrikopoulos NK Kaliora AC Assimopoulou AN Papageorgiou VP Biological activity of some naturally occuring resins, gums and pigments against in vitro LDL oxidation Phytother Res 2003 17 501 507 10.1002/ptr.1185 12748987

[B30] Stocker P Yousfi M Djerridane O Perrier J Amziani R El Boustani S Moulin A Effect of flavonoids from various Mediterranean plants on enzymatic activity of intestinal carboxylesterase Biochimie 2004 86 919 925 10.1016/j.biochi.2004.09.005 15667942

[B31] Ljubuncic P Azaizeh H Portnaya I Coganc U Said O Antioxidant activity and cytotoxicity of eight plants used in traditional Arab medicine in Israel Journal of Ethnopharmacology 2005 99 43 47 10.1016/j.jep.2005.01.060 15848018

[B32] Barra A Coroneo V Dessì S Cabras P Angioni A Characterization of the volatile constituents in the essential oil of *Pistacia lentiscus *L. from different origins and its antifungal and antioxidant activity J Agric Food Chem 2007 55 7093 7098 10.1021/jf071129w 17658828

[B33] Gardeli C Vassiliki P Athanasios M Kibouris T Komaitis M Essentail oil composition of *Pistacia lentiscus *L and *Myrtus communis *L., evaluation of antioxidant capacity of methanolic extracts Food Chemistry 2008 107 1120 1130 10.1016/j.foodchem.2007.09.036

[B34] Tambe Y Tsujiuchi H Honda G Ikeshiro Y Tanaka S Gastric cytoprotection of the non-steroidal anti-inflammatory sesquiterpene, beta-caryophyllene Planta Med 1996 62 469 470 10.1055/s-2006-957942 9005452

[B35] Fernandes ES Passos GF Medeiros R da Cunha FM Ferreira J Campos MM Pianowski LF Calixto JB Anti-inflammatory effects of compounds alpha-humulene and (-)-trans-caryophyllene isolated from the essential oil of Cordia verbenacea Eur J Pharmacol 2007 569 228 236 10.1016/j.ejphar.2007.04.059 17559833

[B36] Passos GF Fernandes ES da Cunha FM Ferreira J Pianowski LF Campos MM Calixto JB Anti-inflammatory and anti-allergic properties of the essential oil and active compounds from Cordia verbenacea J Ethnopharmacol 2007 110 323 333 10.1016/j.jep.2006.09.032 17084568

[B37] Marongiu B Piras A Porcedda S Scorciapino A Chemical composition of the essential oil and supercritical CO2 extract of Commiphora myrrha (Nees) Engl. and of *Acorus calamus *L J Agric Food Chem 2005 53 7939 43 10.1021/jf051100x 16190653

[B38] Adams RP Identification of Essential Oil Component by Gas chromatography/ Quadrupole Mass spectroscopy Allured publishing corporation 2004 Illinois, USA

[B39] Joulain D König WA The atlas of spectral data of sesquiterpene hydrocarbons 1998 E.B.-Verlag, Hamburg

[B40] Folch J Lees M Sloane Stanley GH A simple method for the isolation and purification of total lipides from animal tissues J Biol Chem 1957 226 497 509 13428781

[B41] Banni S Carta G Contini MS Angioni E Deiana M Dessi MA Melis MP Corongiu FP Characterization of conjugated diene fatty acids in milk, dairy products, and lamb tissues J Nutr Biochem 1996 7 150 155 10.1016/0955-2863(95)00193-X

[B42] Melis MP Angioni E Carta G Murru E Scanu P Spada S Banni S Characterization of conjugated linoleic acid and its metabolites by RPHPLC with diode array detector Eur J Lipid Sci Technol 2001 103 617 621 10.1002/1438-9312(200109)103:9<617::AID-EJLT6170>3.0.CO;2-C

[B43] Di Marzo V Goparaju SK Wang L Liu J Bàtkai S Jàrai Z Fezza F Miura GI Palmiter RD Sugiura T Kunos G Leptin-regulated endocannabinoids are involved in maintaining food intake Nature 2001 410 822 825 10.1038/35071088 11298451

[B44] Lowry OH Rosebrough NJ Farr AL Randall RJ Protein measurements with the Folin phenol reagent J Biol Chem 1951 193 265 275 14907713

[B45] De Ley G Nshimyumuremyi JB Leusen I Hemispheric blood flow in the rat after unilateral common carotid occlusion: evolution with time Stroke 1985 16 1 69 73 10.1161/01.STR.16.1.69 3966269

[B46] Coyle P Panzenbeck MJ Collateral development after carotid artery occlusion in Fischer 344 rats Stroke 1990 21 316 321 10.1161/01.STR.21.2.316 2305409

[B47] Garcia MC Ward G Ma YC Salem N Kim HV Effect of docosahexaenoic acid on the synthesis of phosphatidylserine in rat brain in microsomes and c6 glioma cells J Neurochem 1998 70 24 30 9422343 10.1046/j.1471-4159.1998.70010024.x

[B48] Ahmad A Moriguchi T Salem N Decrease in neuron size in docosahexaenoic acid-deficient brain Pediatr Neurol 2002 26 210 218 10.1016/S0887-8994(01)00383-6 11955929

[B49] Niemoller DT Bazan NG Docosahexaenoic acid neurolipidomics Prostaglandins Other Lipid Mediat 2010 91 85 89 10.1016/j.prostaglandins.2009.09.005 19804838 PMC2905848

[B50] Strokin M Sergeeva M Reiser G Role of Ca^2+^-independent phospholipase A_2 _and n-3 polyunsaturated fatty acid docosahexaenoic acid in prostanoid production in brain: perspectives for protection in neuroinflammation Int J Devl Neurosci 2004 22 7 551 557 10.1016/j.ijdevneu.2004.07.002 15465285

[B51] Ando K Nagata K Yoshida R Kikugawa K Suzuki M Effect of n-3 polyunsaturated fatty acid supplementation on lipid peroxidation of rat organs Lipids 2000 35 401 407 10.1007/s11745-000-538-6 10858025

[B52] Nälsén C Vessby B Berglund L Uusitupa M Hermansen K Riccardi G Rivellese A Storlien L Erkkilä A Ylä-Herttuala S Tapsell L Basu S Dietary (n-3) fatty acids reduce plasma F2-isoprostanes but not prostaglandin F2alpha in healthy humans J Nutr 2006 136 1222 1228 16614408 10.1093/jn/136.5.1222

[B53] Na HK Surh YI peroxisome proliferator-activated receptor gamma (PPARγ) ligands as bifunctional regulators of cell proliferation Biochem Pharmacol 2003 66 1381 1391 10.1016/S0006-2952(03)00488-X 14555212

[B54] Pyper SR Viswakarma N Yu S Reddy JK PPARalpha: energy combustion, hypolipidemia, inflammation and cancer Nucl Recept Signal 2010 8 e002 20414453 10.1621/nrs.08002 PMC2858266

[B55] Kaduce TL Chen Y Hell JW Spector AA Docosahexaenoic acid synthesis from n-3 fatty acid precursors in rat hippocampal neurons J Neurochem 2008 105 1525 1535 10.1111/j.1471-4159.2008.05274.x 18248613

[B56] Chen CT Liu Z Bazinet RP Rapid de-esterification and loss of eicosapentaenoic acid from rat brain phospholipids: an intracerebroventricular study J Neurochem 2011 116 363 373 10.1111/j.1471-4159.2010.07116.x 21091476

[B57] Miettinen S Fusco FR Yrjänheikki J Keinänen R Hirvonen T Roivainen R Närhi M Hökfelt T Koistinaho Spreading depression and focal brain ischemia induce cyclooxygenase-2 in cortical neurons through N-methyl-D-aspartic acid-receptors and phospholipase A2 Proc Natl Acad Sci USA 1997 94 6500 6505 10.1073/pnas.94.12.6500 9177247 PMC21079

[B58] Walton M Sirimanne E Williams C Gluckman PD Keelan J Mitchell MD Dragunow M Prostaglandin H synthase-2 and cytosolic phospholipase A2 in the hypoxic-ischemic brain: role in neuronal death or survival? Brain Res Mol Brain Res 1997 50 165 170 9406931 10.1016/s0169-328x(97)00181-2

[B59] Choi JS Kim HY Chun MH Chung JW Lee MY Differential regulation of cyclooxygenase-2 in the rat hippocampus after cerebral ischemia and ischemic tolerance Neurosci Lett 2006 393 231 236 10.1016/j.neulet.2005.09.074 16253424

[B60] Park MK Kang YJ Lee HS Kim HJ Seo HG Lee JH Chang KC The obligatory role of COX-2 expression for induction of HO-1 in ischemic preconditioned rat brain Biochem Biophys Res Commun 2008 377 1191 1194 10.1016/j.bbrc.2008.10.149 18992713

[B61] Nakayama M Uchimura K Zhu RL Nagayama T Rose ME Stetler RA Isakson PC Chen J Graham SH Cyclooxygenase-2 inhibition prevents delayed death of CA1 hippocampal neurons following global ischemia Proc Natl Acad Sci USA 1998 95 18 10954 10959 10.1073/pnas.95.18.10954 9724811 PMC28002

[B62] Matsuoka Y Okazaki M Zhao H Asai S Ishikawa K Kitamura Y Phosphorylation of c-Jun and its localization with heme oxygenase-1 and cyclooxygenase-2 in CA1 pyramidal neurons after transient forebrain ischemia J Cereb Blood Flow Metab 1999 19 1247 1255 10566971 10.1097/00004647-199911000-00009

[B63] Koistinaho J Koponen S Chan PH Expression of cyclooxygenase-2 mRNA after global ischemia is regulated by AMPA receptors and glucocorticoids Stroke 1999 30 1900 1905 discussion 1905-1906 10.1161/01.STR.30.9.1900 10471443

[B64] Collino M Aragno M Mastrocola R Benetti E Gallicchio M Dianzani C Danni O Thiemermann C Fantozzi R Oxidative stress and inflammatory response evoked by transient cerebral ischemia/reperfusion: effects of the PPAR-alpha agonist WY14643 Free Radic Biol Med 2006 41 579 589 10.1016/j.freeradbiomed.2006.04.030 16863991

[B65] Yamashita A Kunimatsu T Yamamoto T Yoshida K Hypothermic, but not normothermic, ischemia causes a drastic increase in cyclooxygenase-2 immunoreactive granule cells in rat dentate gyrus after 4 hours of ischemic reperfusion Arch Histol Cytol 2007 70 197 205 10.1679/aohc.70.197 18079588

[B66] Candelario-Jalil E Fiebich BL Cyclooxygenase inhibition in ischemic brain injury Curr Pharm Des 2008 14 1401 1418 10.2174/138161208784480216 18537663

[B67] Pellegrini-Giampietro DE Mannaioni G Bagetta G Post-ischemic brain damage: the endocannabinoid system in the mechanisms of neuronal death FEBS J 2009 276 2 12 10.1111/j.1742-4658.2008.06765.x 19087195

[B68] Costa B Conti S Giagnoni G Colleoni M Therapeutic effect of the endogenous fatty acid amide, palmitoylethanolamide, in rat acute inflammation: inhibition of nitric oxide and cyclo-oxygenase systems Br J Pharmacol 2002 137 413 420 10.1038/sj.bjp.0704900 12359622 PMC1573522

[B69] De Filippis D D'Amico A Cipriano M Petrosino S Orlando P Di Marzo V Iuvone T Levels of endocannabinoids and palmitoylethanolamide and their pharmacological manipulation in chronic granulomatous inflammation in rats Pharmacol Res 2010 61 321 328 10.1016/j.phrs.2009.11.005 19931394

[B70] Endocannabinoid Research Group De Filippis D D'Amico A Cipriano M Petrosino S Orlando P Di Marzo V Iuvone T Levels of endocannabinoids and palmitoylethanolamide and their pharmacological manipulation in chronic granulomatous inflammation in rats Pharmacol Res 2010 61 321 328 10.1016/j.phrs.2009.11.005 19931394

[B71] Facci L Dal Toso R Romanello S Buriani A Skaper SD Leon A Mast cells express a peripheral cannabinoid receptor with differential sensitivity to anandamide and palmitoylethanolamide Proc Natl Acad Sci USA 1995 92 3376 3380 10.1073/pnas.92.8.3376 7724569 PMC42169

[B72] Lo Verme J Fu J Astarita G La Rana G Russo R Calignano A Piomelli D The nuclear receptor peroxisome proliferator-activated receptor-alpha mediates the anti-inflammatory actions of palmitoylethanolamide Mol Pharmacol 2005 67 15 19 10.1124/mol.104.006353 15465922

[B73] Schomacher M Müller HD Sommer C Schwab S Schäbitz WR Endocannabinoids mediate neuroprotection after transient focal cerebral ischemia Brain Res 2008 1240 213 220 18823959 10.1016/j.brainres.2008.09.019

